# Severe murine schistosomiasis results from disrupted CD4+ T-cell modulation by immunodominance of a single egg epitope

**DOI:** 10.31744/einstein_journal/2024AO0839

**Published:** 2024-11-12

**Authors:** Eduardo Finger, Thaissa Melo Galante Coimbra, Alessandra Finardi Dastoli

**Affiliations:** 1 Faculdade de Ciências Médicas da Santa Casa de São Paulo São Paulo SP Brazil Faculdade de Ciências Médicas da Santa Casa de São Paulo, São Paulo, SP, Brazil.; 2 SalomaoZoppi Diagnósticos São Paulo SP Brazil SalomaoZoppi Diagnósticos, São Paulo, SP, Brazil.

**Keywords:** Immunodominance, Immunopathology, Major histocompatibility complex, CD4-Positive T-Lymphocytes, Schistosomiasis, Immunomodulation

## Abstract

**Objective:**

This study examined the correlation between immunodominance of the major egg antigen Sm-p40_234-246_, a robust Th1/Th17 anti-egg CD4 T-cell response, and severe liver immunopathology in experimental murine schistosomiasis. It serves as a platform to analyze how varying degrees of immunodominance affect CD4+ T cell modulation and disease outcomes.

**Methods:**

We used a murine model of schistosomiasis to investigate the effects of immunodominance. Infected mice were divided into two groups: one treated with a combination of epitopes targeting immunodominance of the major egg antigen Sm-p40 and the other with a mock mixture of non-immunogenic epitopes. Liver granuloma area, a hallmark of schistosomiasis pathology, was quantified using histological and morphometric analyses. The average granuloma areas between the treated and untreated groups were compared using one-way ANOVA with Tukey's multiple comparison test. Additionally, we isolated CD4+ T cells from mesenteric lymph nodes, stimulated them with specific egg antigens, and collected purified supernatants to assess their signature cytokine secretion profiles for each treatment group.

**Results:**

Results showed that strong immunodominance of a single egg epitope undermines effective CD4+ T-cell modulation, promoting a strongly polarized Th1/Th17 pathogenic response. Conversely, neutralizing this immunodominance produces the opposite restorative effect.

**Conclusion:**

Immunodominance is an important pathogenic component that influences CD4+ T cell modulation in experimental murine schistosomiasis. Moreover, immunodominance can be used to treat these and other important CD4+ T cell-mediated diseases.

## In Brief

A peculiar form of antigen presentation, termed restrictive ligand density, may explain why different subjects react to identical antigenic challenges with either a beneficial healing immune response or a pathologically destructive response. This study shows that restrictive ligand density can be neutralized and used to heal or prevent diseases.

## Highlights

■ Restrictive ligand density can be pathological.
■ Restrictive ligand density can be neutralized.
■ Restrictive ligand density neutralization may the used to heal or prevent disease.

## Graphical abstract

**Figure:**
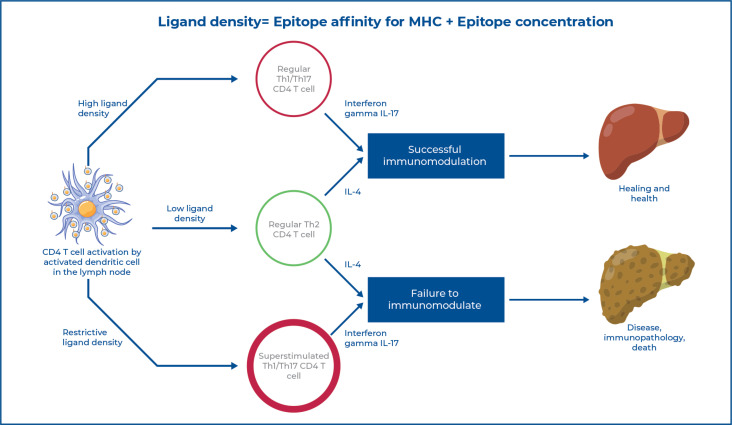

## INTRODUCTION

When faced with an immune challenge, the adaptive immune system strives for the most appropriate strategy to neutralize it with minimal collateral damage.^(1)^ The information required for this is primarily derived from the interaction between antigen-presenting cells (APCs) and CD4+ T cells, where the former provide epitopes, cytokines, and co-stimulation, and the latter process these into immune-challenge-specific responses. For example, Th1 responses target intracellular organisms, Th2 responses target helminths or toxins, and Th17 responses target extracellular organisms. The threat is neutralized if the immune response is appropriate, otherwise resulting in prolonged illness and immunopathology.

The process of adjusting the immune strategy to a challenge is known as modulation. Although not entirely understood, modulation involves dynamic interactions among at least six components: innate immune signals,^(2)^ epitope concentration,^(3)^ epitope affinity for the major histocompatibility complex (MHC),^(4)^ T-cell receptor (TCR) affinity for the MHC/epitope ligand,^(5)^ co-stimulation^(6,7)^ and the local immune environment.^(8)^

Immunodominance (ID), the phenomenon where the immune system targets a limited number of highly immunogenic epitopes from an antigen (referred to as dominant or immunodominant epitopes), also plays a critical role in the efficiency of immune responses. A robust immune response against a key dominant epitope often leads to the effective clearance of that immune challenge. While the exact mechanisms linking ID to modulation remain to be fully elucidated, both are influenced by three common components: epitope availability, epitope affinity for the MHC and epitope recognition by the TCR.

Murine schistosomiasis egg-induced liver immunopathology (SELI) is a useful and reliable model to study modulation,^(9)^ and elucidate the role of its various components.^(10,11)^ In this model, infection with *Schistosoma mansoni* initially elicits a mild Th1 response until four weeks post-infection (WPI), when mature schistosomes begin to lay eggs. Some eggs are trapped in the portal spaces of the liver, and elicit a CD4+ T cell-mediated granulomatous response.^(12)^ Infected C57BL/6 (H-2B) and BALB/c (H-2D) mice are low-pathology strains because they modulate the initial Th1 anti-egg response to a Th2 response, resulting in small, well-defined perioval granulomas and minor liver damage. Conversely, CBA/J and C3H/HeN mice (both H-2K) represent high-pathology strains because upon contact with eggs, their initial Th1 responses accelerate and expand into a stronger, polarized Th1/Th17 response, producing large, infiltrative, highly inflammatory perioval granulomas, widespread liver damage, and a severe and frequently lethal disease.

Analysis of the CD4+ T cell response against isolated purified Schistosoma egg antigens showed that high- and low-pathology strains can be segregated according to their response to the major Sm-p40 egg antigen, specifically its immunodominant epitope, Sm-p40_234-246_. CD4+ T cells from high-pathology strains respond to Sm-p40 with strong Th1/Th17 polarization, similar to that observed in experimental infection; however, CD4+ T cells from low-pathology strains remain unresponsive.^(13,14)^ This observation suggests that the anti-Sm-p40 CD4+ T cell response may be pivotal for the exacerbation of the strong Th1/Th17 polarized reaction that precedes and correlates with the development of severe pathology.^(15)^

Sm-p40 is a 354 amino acid glycoprotein that comprises 10% of Schistosoma egg proteins.^(16)^ The I-A^k^ MHC haplotype restricts Sm-p40 to two subdominant epitopes (Sm-p40_179-208_ and Sm-p40_279-308_) and a major dominant epitope (Sm-p40_234-246_).^(14)^ This dominant epitope is the target of the Vα11.3β8^+^ CD4+ T cell clonotype, a specific clonotype highly prevalent in the egg induced CD4+ T cell repertoire in high pathology strains, but not in low pathology ones.^(17)^ The I-A^b^ haplotype restricts Sm-p40 to five epitopes and I-A^d^ haplotype to 18 epitopes, none of which are dominant (Table 1).

**Table 1:** Number of Sm-p40 epitopes predicted to be presented by different MHC II haplotypes per the RANKPEP algorithm

Haplotype	Binding threshold*	Number of Sm-p40 epitopes presented
I-A^k^	14.17	2
I-A^b^	9.52	5
I-A^d^	7.1	18
I-A^s^	12.65	1

* Minimum score required for an epitope to be presented by the MHC molecule.

This suggests that the failure to modulate, and consequently, the development of high pathology in murine schistosomiasis, depends on the CD4+ T cell response to one major dominant epitope. However, how could the CD4+ T-cell response to a single epitope determine the outcome of a complex disease such as schistosomiasis?

One possible explanation is ligand density (LD), a concept proposed by Bottomly et al.^(4,18–20)^ which suggests that MHC II epitope loading and presentation are competitive processes dependent on epitope concentration and affinity for MHC II. If no individual epitope prevails, the antigen presentation includes many epitopes, each being presented in low numbers, which constitutes a low LD, leading to a Th2 response. Conversely, if one epitope outcompetes the others in terms of concentration or affinity, it occupies a larger portion of available MHC II molecules, producing a high LD, that elicits a Th1 response.^(19,21)^ In this scenario, Sm-p40 epitomizes the perfect storm against the effective modulation of the anti-egg CD4+ T cell response in H-2K strains because it is the most abundant antigen within eggs, and its dominant epitope exhibits a particularly high affinity for I-A^k^, a more stringent MHC II haplotype.^(14, 22)^

Consequently, Sm-p40_234-246_ achieves an exceptionally high LD in the I-A^K^ strains, which generates an overwhelming pro-Th1/Th17 stimulus that overrides any modulation efforts. This could explain why despite secreting several-fold more Th2 cytokines than low pathology strains, high-pathology strains still develop severe liver immunopathology.

## OBJECTIVE

This study analyzed the effect of restrictive immunodominance (the situation where one epitope overwhelmingly dominates the MHC II presentation, thus producing an exceedingly high ligand density) on CD4+ T-cell modulation and its immunopathogenic outcomes.

## METHODS

### Mice and infection

CBA and SJL mice were acquired from the *Universidade Estadual de Campinas*, and C57BL/6 mice were purchased from *Universidade Federal de São Paulo*. The mice were housed and monitored at our dedicated animal research facility. This study was approved by the Institutional Committee for Ethics in Animal Experimentation of *Faculdade de Ciências Médicas da Santa Casa* (#140).

For each experiment, 10 female mice were infected at 6 weeks of age using intraperitoneal injection of 150 *S. mansoni* cercariae and euthanized at 8 WPI.

### Morphometric and statistical analysis

Mouse livers were collected in 10% formaldehyde, processed into 5*μ*m thick sections, stained with hematoxylin and eosin, and examined using an Eclipse 50 microscope equipped with a digital image acquisition system (Nikon Instruments, Melville, NY). Granuloma areas were measured using the NIS-Elements D 3.0 imaging software (Nikon Instruments) for granulomas containing a single visible central egg. Statistical analysis was performed using a one-way ANOVA with Tukey's multiple comparison test (GraphPad Prism 6, La Jolla, California, USA).

### Cell culture, CD4+ T-cells, and APC purification and preparation

Immediately post-euthanasia, mesenteric lymph nodes (MLN) were aseptically removed and manually dissociated into cell suspensions in cRPMI [RPMI1640 (Hyclone Laboratories, Logan, UT), supplemented with 10% FCS (Hyclone), 4 mM L-glutamine, 80 U/ml penicillin and 80 mg/ml streptomycin (Hyclone), 1 mM sodium pyruvate (Hyclone), 10 mM HEPES, 1x non-essential amino acids (Sigma, St. Louis, MO) and 6 x10^-5^ M 2-ME].

This suspension was treated with Tris-ammonium chloride erythrocyte lysis buffer for 15 min, quenched with cRPMI, washed thrice, tested for cell concentration and viability, and then diluted for magnetic purification of CD4+ T-cells according to the manufacturer's instructions provided with the CD4-T-cell-isolation kit (Miltenyi Biotech, Auburn, CA).

APCs were prepared from spleens of at least 2 uninfected mice of the CBA, C57BL/6 or SJL strains using the same method and then treated for 20 minutes at 37°C with mitomycin C 50 *μ*g/ml (Sigma).

### Epitope selection and synthesis

The evaluation and selection of haplotype-specific dominant epitopes were performed *in silico* by submitting the sequences of schistosome egg proteins published into the ENTREZ and SchistoDB databases for analysis using the RANKPEP algorithm.^(23)^

The epitopes selected for Sm-p40_234-246_ restrictive immunodominance (RI) neutralization experiments were synthesized by Life Technologies (Grand Island, NY, USA). Cell Polarity Protein (CPP)_1380-1397_, the RI-inducing epitope for the H-2^b^ haplotype, was synthesized at *Universidade Federal de São Paulo*.

### Neutralization or induction of RI

**Figure 1:**
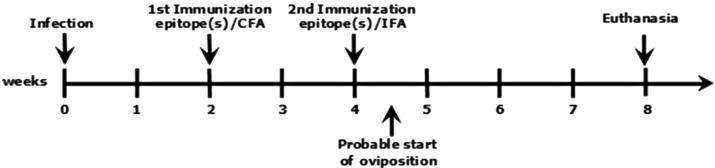
Schematic illustration of the experimental method for the neutralization of Sm-p40_234-246_ RI in CBA mice or induction of CPP_1380-1397_ RI in C57BL/6 mice

To neutralize Sm-p40_234-246_ RI, CBA mice were subcutaneously administered a mixture containing 2 *μ*M of each of the 4 competitor epitopes for immunization (a total of 8 *μ*M per mouse per immunization) (Table 2, Figure 1). The immunization mixture was diluted in phosphate buffered saline (PBS) before emulsification with an adjuvant. The first immunization was administered 2 WPI, emulsified in complete Freund's adjuvant, followed by a second immunization 4 WPI with incomplete Freund's adjuvant. Restrictive immunodominance induction in C57BL/6 mice was performed using a similar method, however, 5*μ*M CPP_1380-1397_ was used instead.

**Table 2:** MHCII/Epitope affinity as predicted by the RANKPEP algorithm

Epitope	Score*	Sequence
I-A^k^ MHCII haplotype		
Sm-p40_234-246_	17.59†	PKSDNQIKAVPAS
PEPCK_341-365_	15.14	VAPGTNVKTNPNAMA
SmE16_110-125_	14.91	MDIDQNSLRNWMTQN
Pré-Alb_268-282_	22.91	ECCHGDLLECADRA
Pré-Alb_333-345_	19.35	FVEDKEVCKNYAEAK
I-A^b^ MHCII haplotype		
CPP_1380-1397_	26.62‡	SRTNYAGPTCNIPLCPPN

* The higher the score, the higher the affinity between the epitope and the MHCII molecule;

† The maximum possible score for an I-A^k^ restricted epitope is 39.90. Epitopes with a score <14.17 are unlikely to be presented;

‡ The maximum possible score for an I-A^b^ restricted epitope is 35.63. Epitopes with a score <9.52 are unlikely to be presented.

### Cytokine analysis

For cytokine profile analysis, 1.5 × 10^6^ CD4+ T-cells were co-cultured with an equal number of syngeneic APCs at 37°C, with 5% CO_2_, with or without addition of soluble egg antigens (SEA), Sm-40 and the mixture of non-antigenic peptides. Concanavalin A (Sigma-Aldrich) was used as a control.

After 72 h, supernatants were collected, filtered using a 0.2mm syringe filter (Millipore, Darmstadt, Germany), and analyzed using a cytometric bead array (CBA) analysis kit (BD, Carlsbad, CA), according to the manufacturer's instructions, using a FACSCalibur cytometer (BD). Cytokines analyzed included IL-4, IL-10, Interferon-g, and IL-17.

## RESULTS

### Sm-p40_234-246_ RI neutralization prevents severe SELI in CBA mice

To assess whether neutralizing Sm-p40_234-246_ RI could effectively modulate disease outcomes and mitigate severe liver immunopathology, we immunized infected CBA mice (Figure 1) with a mixture of four synthetic epitopes.

These epitopes were carefully selected for their presence within egg proteins, and their affinity for I-A^k^ was comparable to that of Sm-p40_234-246_ (Table 2). Consequently, APCs could present five epitopes instead of one, mirroring the epitope presentation observed in low-pathology C57BL/6 mice (Table 1).

**Figure 2:**
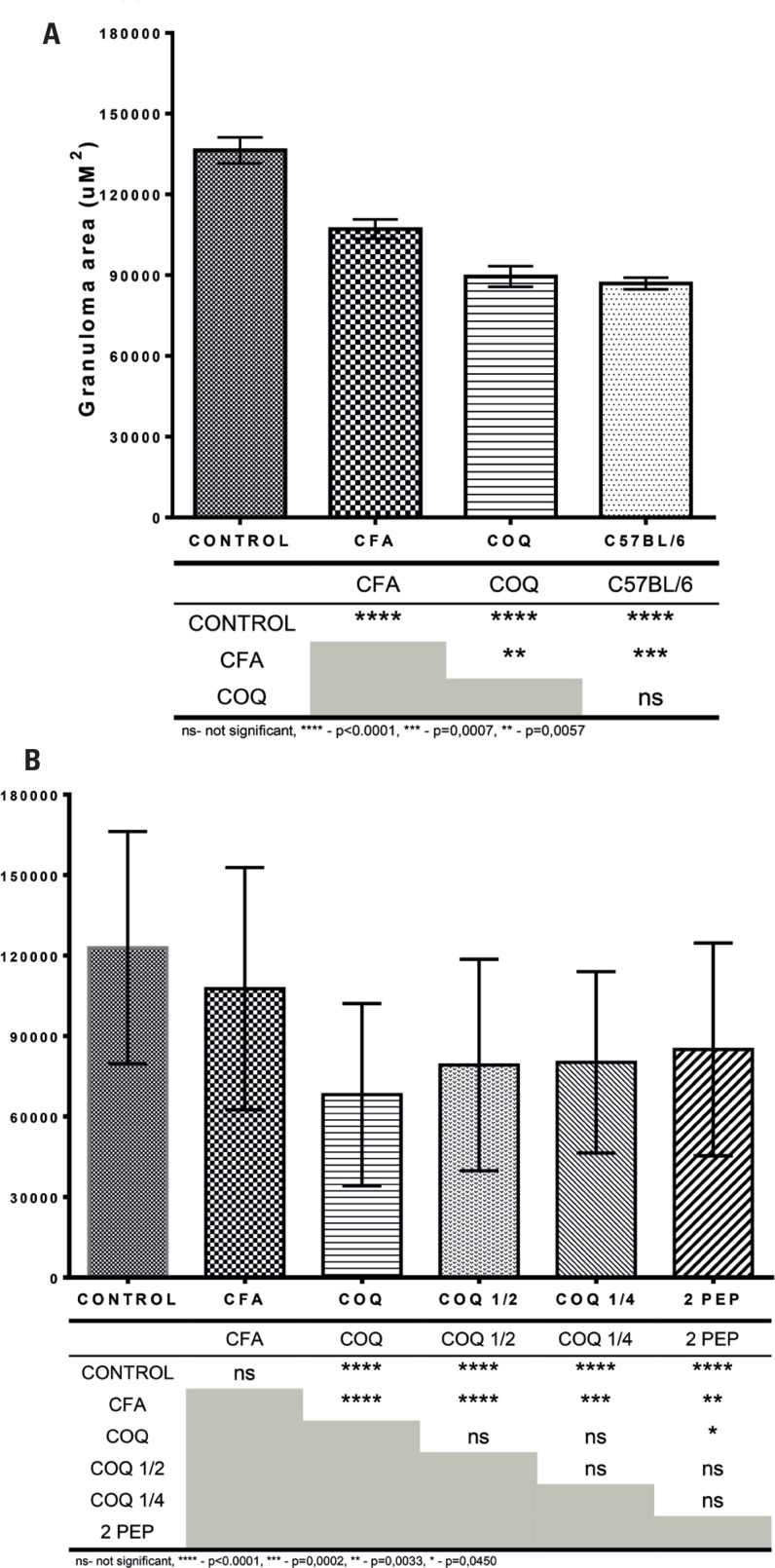
Morphometric analysis of egg-induced liver granulomas showing that Sm-p40_234-246_ RI neutralization reduces the severity of SELI in Schistosoma-infected female CBA mice. A) Comparison of SELI in untreated CBA mice (CONTROL), mice immunized solely with adjuvant (CFA), mice that were subjected to Sm-p40_234-246_ RI neutralization (COQ) and untreated low pathology C57BL/6 mice; B) Comparison of effects of complete Sm-p40_234-246_ RI neutralization (COQ), with half the dose of the epitope mixture (COQ ½), a quarter of the dose (COQ ¼) or a different composition of the mixture that contained 2 epitopes instead of 4 (2 PEP). The table below the graphs shows the statistical differences between the test groups. Results are representative of 6 different experiments. No test group contained less than 3 mice or 78 granulomas. Use of a mock mixture without affinity for I-A^k^ produced no change in CBA SELI. ^a^ – In this experiment, the SELI of the CFA differed significantly from that observed in the CONTROL group. This is not the typical outcome for this group and is compensated by the fact that group COQ also differs significantly from group CFA

**Figure 3:**
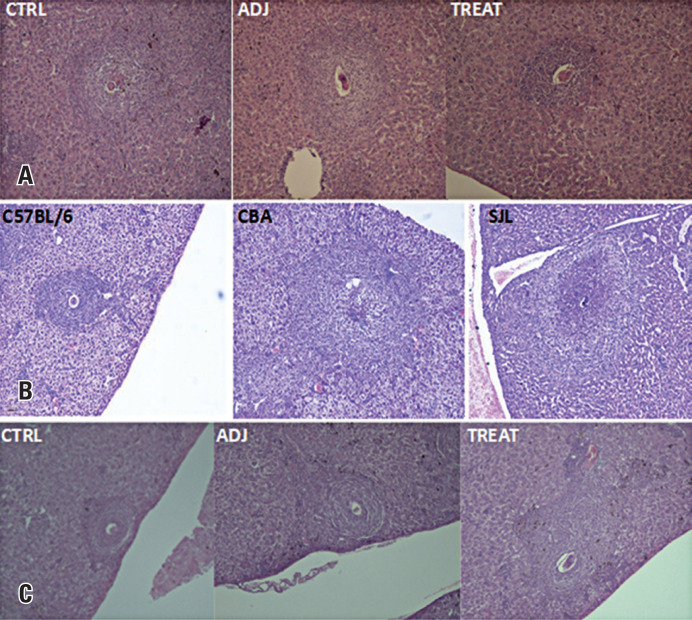
Comparative liver immunopathology of the three experimental groups. A) Sm-p40_234-246_ RI neutralization showing representative granulomas from the untreated CBA controls (CTRL), CBA mice treated with adjuvant only (ADJ) and CBA mice that were subjected to RI neutralization (TREAT); B) Representative granulomas in mouse strains whose MHC II haplotypes produce Sm-p40 RI (CBA and SJL) or not (C57BL/6); C) Representative granulomas from untreated C57BL/6 mice (CTRL), C57BL/6 mice treated with adjuvant alone (ADJ), or C57BL/6 mice treated with the RI inducing peptide CPP_1380-1397_ (TREAT)

Morphometric analysis showed that RI neutralization significantly reduced severe liver immunopathology in CBA mice in a dose-dependent manner. Remarkably, this mitigation resulted in liver immunopathology in CBA mice similar to that in C57BL/6 mice, which typically exhibit low pathology (Figure 2 and 3). Notably, administration of a mock mixture containing four random peptides lacking affinity for I-A^k^ did not alter the severity of liver immunopathology in CBA mice (data not shown).

### Non-H-2K strains subject to Sm-p40 RI develop severe SELI

To ascertain whether Sm-p40 RI exacerbates liver immunopathology, we analyzed Sm-p40 restriction patterns across various MHC II haplotypes. Our analysis revealed that the Sm-p40 restriction in the I-A^s^ haplotype was even more stringent than that in the I-A^k^ haplotype (Table 1). Consequently, we hypothesized that H-2S SJL mice infected with *Schistosoma mansoni* would exhibit liver immunopathology similar to that observed in CBA mice.

**Figure 4:**
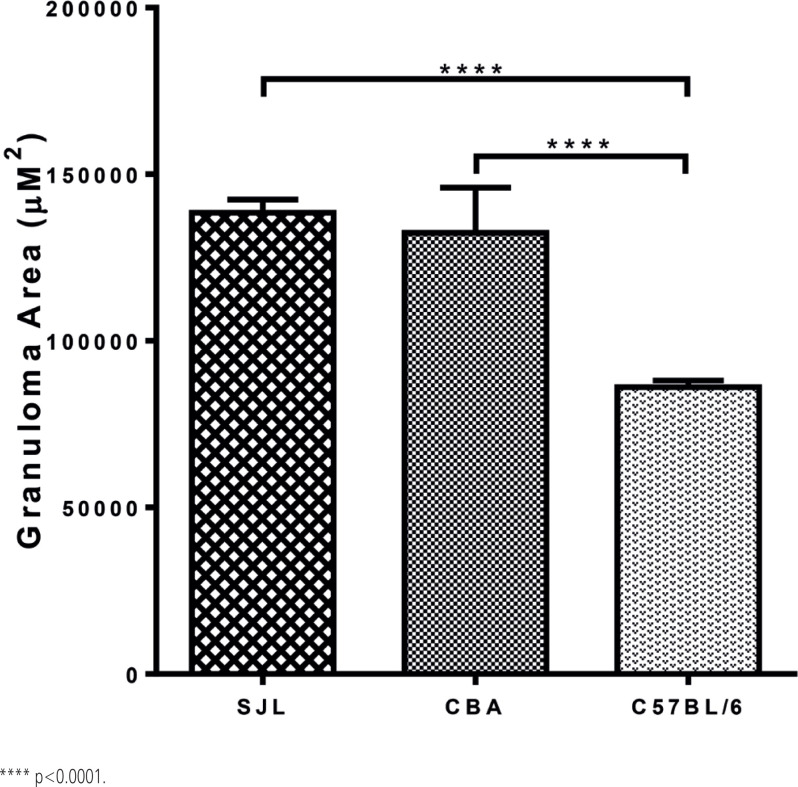
Morphometric analysis of egg-induced liver granulomas from schistosome infected SJL, CBA, and C57BL/6 mice showing that, in a different MHC II haplotype, severe SELI correlates with restrictive immunodominance of Sm-p40. Results are representative of four different experiments. No test group contained less than 3 mice or 176 granulomas

Morphometric analysis verified our hypothesis that infected SJL mice exhibited liver immunopathology with severity comparable to that of CBA mice (Figure 3 and 4), with a trend towards progression to severe disease, although this difference was not statistically significant.

### Experimentally induced RI leads to severe SELI in C57BL/6 mice

**Figure 5:**
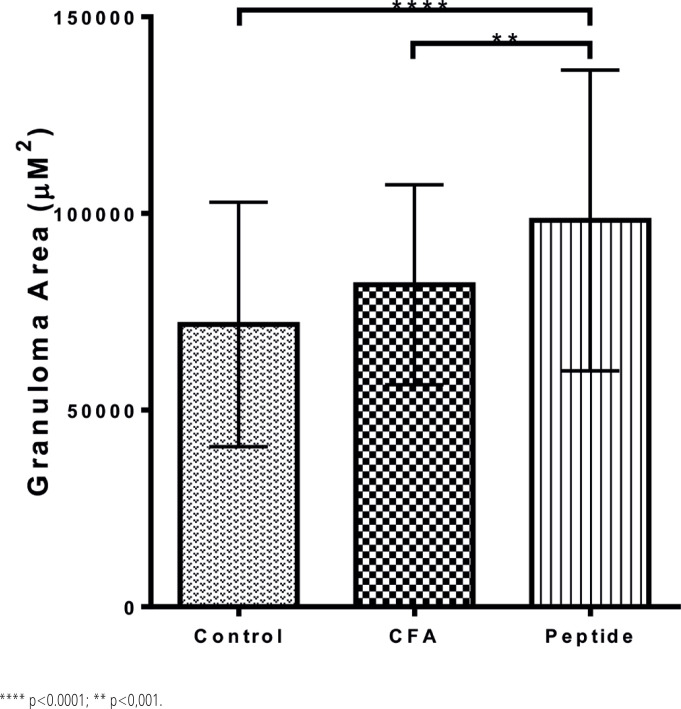
Morphometric analysis of egg-induced liver granulomas from schistosome infected C57BL/6 untreated mice (Control), immunized solely with adjuvant (CFA), or adjuvant plus CPP_1380-1397_ (Peptide), a synthetic epitope capable of inducing restrictive immunodominance in I-A^b^ mice, showing that severe SELI correlated with induction of RI by a single epitope

To investigate whether a single epitope could pathogenically influence an otherwise adequate anti-egg CD4+ T-cell response, we searched the *Schistosoma mansoni* genome for the highest-ranked epitope with affinity for I-A^b^ expressed within an egg protein (Table 2). Immunization of C57BL/6 mice (Figure 1) with this RI-inducing epitope significantly aggravated the liver immunopathology, as shown using morphometric analysis (Figure 3 and 5).

### Sm-p40_234-246_ RI neutralization reduces cytokine polarization in infected CBA mice

If Sm-p40_234-246_ RI drives the pro-Th1/Th17 response observed in severe schistosomiasis, its neutralization should reduce the production of Th1/Th17 signature cytokines, namely interferon gamma (IFN-g) and IL-17.

To test this, we analyzed *ex vivo* cytokine production in CD4+ T cells purified from the mesenteric lymph nodes of CBA mice 8 weeks post-infection, with or without RI neutralization.

**Figure 6:**
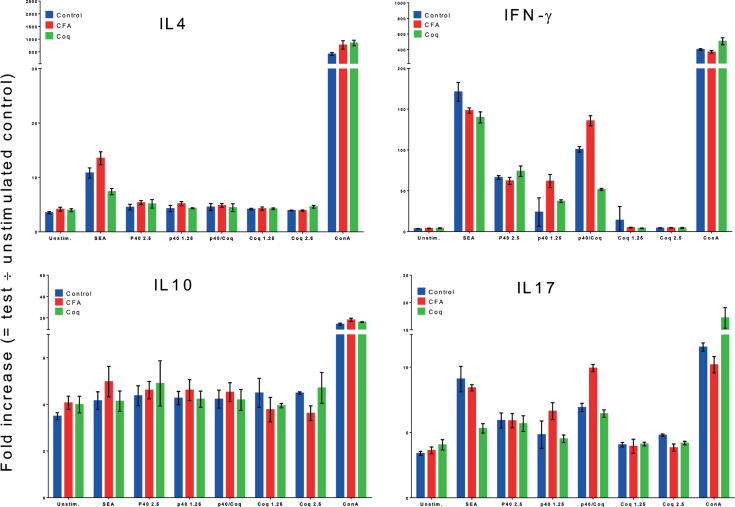
Analysis of *ex vivo* production of IL4, IL10, IFN-**g** and IL17 by CD4+ T-cells purified from the mesenteric lymph nodes of CBA mice 8 wpi, subjected to standard RI neutralization protocols, stimulated using 25 *μ*g/ml of soluble egg antigens (SEA), 2.5 *μ*M and 1.25 *μ*M Sm-p40 (p40), 2.5 *μ*M and 1.25 *μ*M RI neutralization cocktail (Coq), a mixture of both at 1.25 *μ*M each (p40/Coq) and 10 *μ*g/ml concanavalin A (ConA). As previously described, Sm-p40 does not elicit IL4 or IL10 responses in CBA mice, however, elicits significant IFN-**g** and IL17 production, curtailed by RI neutralization (Coq) compared to mice administered only adjuvant (CFA)

Our results consistently revealed lower levels of IFN-g and IL-17 against Sm-p40 in mice treated for RI neutralization, compared to those immunized with adjuvant alone. Notably, Sm-p40 did not elicit Th2 cytokines in the CBA mice, as reported previously ^(9)^ (Figure 6).

### Is RI relevant for human CD4 T-cell mediated diseases?

To assess the relevance of RI in human CD4+ T-cell mediated diseases, we conducted RANKPEP restriction analysis for three diseases characterized by dominant CD4+ T-cell mediated pathogenesis and strong associations with specific MHC II haplotypes and known or suspected eliciting antigens: Lyme disease (OspA/DRB1*0401),^(24)^ celiac disease (gliadin/DQ2.5)^(25)^ and multiple sclerosis (myelin/DRB1*1501/02)^(26)^ (Table 3).

**Table 3:** MHCII specific restriction analysis of antigens correlated with CD4 T-cell mediated autoimmune diseases using the RANKPEP algorithm

CD4 T-cell mediated disease	Eliciting antigen	MHCII haplotype restriction	Number of competitive epitopes	MHCII affinity score of fittest epitopes relative to the maximum score£ %	MHCII affinity ratio between the 1^st^ and 2^nd^ fittest epitopes§
Lyme disease	OspA	DRB1*0401	10‡	52.52	2,50
Celiac disease	gliadin	DQ2.5	1	47.78	1.28
Multiple sclerosis	myelin≠	DRB1*1501	10†	96.81	2.34
		DRB1*1502	3	81.03	6.27

‡ Although RANKPEP predicts 10 possible epitopes, several of them are overlaps of the same epitope; therefore, there are only 5 unique epitopes;

† Although RANKPEP predicts 10 possible epitopes, several of them are overlaps of the same epitope; therefore, there are only 6 unique epitopes;

£ Calculated by dividing the MHCII affinity score of the highest scored epitope by the maximum predicted score for that haplotype;

§ Calculated by dividing the MHCII affinity score of the highest scored epitope by the affinity score of the 2^nd^ highest scored unique epitope;

≠ Not demonstrated; however, speculated to be one of the possible autoantigens involved in multiple sclerosis.

Our analysis revealed that, in these diseases, the highest-ranked epitope for the MHC haplotype either monopolized the MHC presentation pathway or significantly outperformed the second-ranked epitope, meeting the criteria for RI establishment. Given their autoimmune origins, antigen availability (stoichiometry) poses no limitations, making RI a potential driver of pathogenesis.

Collectively, our analysis of these three autoimmune diseases suggests that the RI mechanisms observed in murine schistosomiasis may extend to human immunopathogenesis, highlighting the role of RI in CD4+ T cell-mediated diseases.

## DISCUSSION

In the late 1800s, the advent of the paradigm of antigens as elicitors and modulators of the immune response established modern immunology. Despite decades of research, fully unraveling the mechanisms by which antigens influence immune responses remains a complex challenge. However, fundamental principles such as immunodominance (ID) have been established.

The significance of ID was first recognized by virologists, who noted that minimal changes to a dominant epitope could transform self-limited viral diseases into chronic conditions.^(27)^ In contrast, similar evidence for CD4+ T cell-mediated processes did not confer ID an equivalent role in MHC II-driven immune responses. Nonetheless, numerous examples have emerged over the years, in which a single dominant epitope presented to a particular CD4+ T cell clonotype by a specific MHC II haplotype leads to a disproportionately polarized response that resists immunomodulation and generates disease. Examples include murine leishmaniasis,^(8)^ experimental allergic encephalomyelitis,^(28)^ Lyme disease,^(24)^ respiratory syncytial virus pneumonitis^(29)^ and schistosomiasis.^(17)^

Our study aimed to deconstruct this process and test whether the unique association between a disproportionately dominant epitope and a specific MHC II haplotype, termed restrictive immunodominance (RI), could explain the pathogenic transformation of the CD4+ T cell response. We found that a) neutralizing RI prevents disease, b) inducing RI promotes disease, and c) antigens proven or suspected to produce human disease fulfill the conditions required to produce RI.

Our results suggest that RI, an exceptional antigen-presenting state, might cause failure of CD4+ T cell immunomodulation and its transformation into a pathogenic response. According to this rationale, the primary difference between schistosomiasis and diseases resulting from a "rogue" CD4+ T-cell response is the location of the target epitope, whether in the liver, pancreas, skin, intestine, or brain. Therefore, correcting RI could potentially prevent diseases, such as type I diabetes, pemphigus, celiac disease, and multiple sclerosis.

This insight also sheds light on the long-standing, however, poorly understood association between MHC genes and autoimmune diseases. The disease results from the domination of the MHC presentation pathway by an inexhaustible antigen supply with a disproportionately high affinity for MHC molecules, creating an intense and polarized CD4+ T-cell response that resists modulation.

Restrictive immunodominance neutralization can be used even if the identity of the eliciting epitope is unknown. To counter RI, its establishment must be prevented, which only requires characterization of the MHC haplotype. This strategy may be particularly helpful for preventing rejection during organ transplantation.

Mechanistically, RI neutralization may function by priming a naïve CD4+ T cell population into terminally differentiated Th2 egg-specific cells. At the start of oviposition, these cells migrate to the egg/granuloma interface and counteract the polarizing effects of Sm-p40_234-246_ RI. Supporting evidence for the role of *ad-hoc* pre-primed CD4+ T-cell populations is provided by the work of Julia et al., showing that the strong IL-4 response of Vα8β4+ CD4 T-cells upon Leishmania infection originated from memory cells primed in the intestine by the microbiota.^(30)^

## CONCLUSION

This study demonstrated that restrictive immunodominance significantly influences the severity of murine schistosomiasis by modulating the CD4+ T cell response to a single dominant egg epitope, Sm-p40_234-246_. Neutralizing the restrictive immunodominance of this epitope in high-pathology CBA mice ameliorated the severe liver immunopathology, resulting in granuloma formation and liver damage comparable to those observed in low-pathology C57BL/6 mice. Conversely, inducing restrictive immunodominance in low-pathology C57BL/6 mice through immunization with an epitope with high affinity for I-Ab exacerbates liver immunopathology, highlighting the pivotal role of restrictive immunodominance in disease progression. Additionally, the severe pathology observed in SJL mice, which carries the I-As haplotype and exhibits an even more stringent restriction of Sm-p40 than I-Ak, further supports the impact of restrictive immunodominance on disease severity.

These results indicate that restrictive immunodominance disrupts effective immunomodulation by promoting a polarized Th1/Th17 response, as shown by elevated levels of signature cytokines IFN-g and IL-17 in high pathology strains, and their downmodulation upon addition of the restrictive immunodominance neutralization mixture.

This finding underscores failure of the immune system to modulate towards a Th2 response, which is characteristic of low-pathology strains, and suggests that a single immunodominant epitope can derail modulation, consequently driving severe immunopathology in schistosomiasis.

Furthermore, analysis of human diseases characterized by dominant CD4+ T-cell-mediated pathogenesis, such as Lyme disease, celiac disease, and multiple sclerosis, suggests that restrictive immunodominance might contribute similarly to their pathogenesis.

These findings suggest that the mechanisms of restrictive immunodominance observed in murine schistosomiasis may be applicable to human CD4+ T cell-mediated diseases, offering potential therapeutic avenues for preventing or mitigating disease severity by targeting and neutralizing restrictive immunodominance.

In summary, this study identified restrictive immunodominance as a key pathogenic component of schistosomiasis and other CD4+ T cell-mediated diseases. By elucidating the role of restrictive immunodominance in immune modulation and disease outcomes, our findings provide a foundation for the development of novel therapeutic strategies for neutralizing restrictive immunodominance to prevent or ameliorate severe immunopathology.
